# High-performance silver nanowires transparent conductive electrodes fabricated using manufacturing-ready high-speed photonic sinterization solutions

**DOI:** 10.1038/s41598-021-03528-w

**Published:** 2021-12-17

**Authors:** Luis Felipe Gerlein, Jaime Alberto Benavides-Guerrero, Sylvain G. Cloutier

**Affiliations:** Department of Electrical Engineering, École de technologie supérieure, Montréal, H3C 1K3 Canada

**Keywords:** Engineering, Electrical and electronic engineering, Electronic devices, Nanowires

## Abstract

On the long road towards low-cost flexible hybrid electronics, integration and printable solar energy harvesting solutions, there is an urgent need for high-performance transparent conductive electrodes produced using manufacturing-ready techniques and equipment. In recent years, randomly-distributed metallic nanowire-based transparent mesh electrodes have proven highly-promising as they offer a superb compromise between high performances and low fabrication costs. Unfortunately, these high figure-of-merit transparent mesh electrodes usually rely heavily on extensive post-deposition processing. While conventional thermal annealing yields good performances, it is especially ill-suited for deposition on low-temperature substrates or for high-throughput manufacturing solutions. Similarly, laser-induced annealing severely limits the processing time for electrodes covering large surfaces. In this paper, we report the fabrication of ultra high-performance silver nanowires-based transparent conductive electrodes fabricated using optimized manufacturing-ready ultrafast photonic curing solutions. Using conventional indium tin oxide (ITO) as our benchmark for transparent electrodes, we demonstrate a 2.6–2.7 $\times$ performance gain using two different figure-of-merit indicators. Based on these results, we believe this research provides an ideal manufacturing-ready approach for the large-scale and low-cost fabrication of ultra high-performance transparent electrodes for flexible hybrid electronics and solar-energy harvesting applications.

## Introduction

New critical applications ranging from internet-of-things (IoT) and biomedical sensors to wearable and energy-harvesting devices are currently forcing the consumer electronics industry to revise its manufacturing paradigms. To meet this new and exponentially-growing demand, the industry is seeking new flexible hybrid electronic solutions using printable circuits and components. Many of those key applications including solar cells^1,2^, light emitting devices^3^, haptic displays and touch screens^4^, wearable electronics^3,5,6^ and biomedical sensors^7^ usually require a high-performance transparent conductive electrode (TCE) technology compatible with high-speed manufacturing techniques and equipment. Ideally, a printable solution-based TCE solution would be compatible with traditional rigid substrates, but also with flexible substrates using polyethylene or polyimide, amongst others^8,9^. Unfortunately, extensive post-treatment steps are usually necessary to achieve the best TCE performance^10–13^. This significantly affects the manufacturing capabilities and costs.

Today, the most established TCE technology is by far the Sb-doped indium oxide (or so-called ITO) glass films^14,15^. However, brittleness, material availability and high processing costs make it ill-suited for many emerging applications when it comes to large-scale manufacturing^15,16^. While they can offer some advantages, other doped metal-oxides including aluminum-doped zinc oxide (AZO) and fluorine-doped tin oxide (FTO)^15^ also suffer from structural and manufacturing limitations akin to ITO’s. Conductive conjugated polymer solutions can be more easily processed; however, they are also limited when it comes to performances and large scale manufacturing^17^. Besides their organic nature makes them prone to oxidation and photodegradation, which requires additional post-treatment steps^18,19^. More recently, graphene-, nanotube- or nanowire-based conductive mesh electrodes have proven a promising alternative to oxide-based TCE films.

Metallic nanowire-based mesh electrodes are especially promising contenders to replace ITO as the next-generation TCE standard. This is largely due to their facile and low-cost processing in ambient conditions, low sheet resistance, high transparency and flexibility^20,21^. Indeed, they show low sheet resistance (R$_{sh}$), exceeding 90% of optical transparency^22–24^. As such, they have become especially attractive for low-cost printable photovoltaic devices architectures^25^. For haptic displays and sensing applications^7,21^, requirements for sheet resistance are on the order of 500 $\Omega /{\text{sq.}}$^26^. As such, metallic nanowire-based TCEs are also well suited for these applications. They also provide the added benefit of a better tolerance to mechanical bending for flexible device integration^7,27^.

### State-of-the-art

Traditionally, percolated silver nanowire mesh electrodes require thermal annealing above 300 $^\circ$C in order to properly connect the nanowires together and achieve the best performances^28^. The conventional thermally-induced sintering process imposes a stringent time-to-temperature trade-off restriction, such that lower annealing temperatures generally require much longer processing times^29^. Previously, incremental densification of the silver nanowire-based film through pressure rolling clearly established the direct relationship between the post-processing treatment and the electrodes’ improved electrical and mechanical properties^9,30,31^. Indeed, these lengthy post-processing treatments can successfully yield spectacular sheet resistance values, under 14 $\Omega /{\text{sq.}}$sq., together with an improved mechanical bending capability^32^. Unfortunately, imposing such high temperature treatments on most sensitive low-temperature substrates will also yield severe degradation. In contrast, reducing the process temperature will necessarily require increasing the processing time and significantly slow-down the manufacturing process^29^.

As an alternative to conventional thermal annealing, light-assisted post-processing such as laser-induced sintering^8^ and broadband pulsed-light or high-intensity photonic curing^33–37^ can allow for room-temperature processing of a variety of nanoparticles ranging from metallic^35,38^ to chalcogenides^37^ and semi-conducting^39,40^ aggregates. It also enables a wider variety of low-cost and low-temperature substrates^34^. Carefully-optimized photonic curing can deliver results akin to conventional thermal annealing process, but in much faster processing times^34,41^. This is done by exciting plasmon resonance-induced heating, without damaging the underlying substrate. With silver nanowires, visible light excites a plasmonic resonance and heat dissipation occurs rapidly along their longitudinal axis thus facilitating their interconnection^41^. Meanwhile, thermal equilibrium can be reached within milliseconds within the substrate^33,34^. Moreover, the heat can’t reach the opposite side of the substrate, thus preventing unwanted warping, expansion or cracks in the substrate^37^. As a result, functional materials can be processed at temperatures much higher than those tolerated by the underlying substrates. Therefore, a small number of high fluence, short pulses could potentially densify the film of silver nanowires on a low-temperature substrate, hopefully reaching sheet resistance values comparable to values obtained after several minutes, or hours, of conventional thermal annealing.

It was previously established that good conductivity can be obtained using photonic curing with low-energy density fluence, but at the cost of using an elevated number of pulses^36^. Indeed, excitation with over 150 pulses can already yield sheet resistance values under 0.15 $\Omega /{\text{sq.}}$ for films composed of silver nanoparticles of 40 nm in diameter^36^. In contrast, reducing the number of pulses usually requires very thick silver nanoparticle films. Indeed, resistivity down to 6.9 $\upmu \Omega /{\text{sq.}}$ was previously achieved using only 6 pulses, but only for silver films over 4.35 $\upmu$m in thickness^42^.

Two key parameters play a definite role in the fabrication of any high-performance transparent conductive electrodes, (1) the optical transparency (T) and (2) the sheet resistance (R$_{sh}$). These key parameters are commonly combined using two different figures-of-merit indicators. The first one can be defined as the electrical to optical conductivity ratio^43^ $\sigma _{DC}/\sigma _{OP}$, while the second represents the figure of merit for transparent conductive materials^44,45^ $\Phi _{TC}$. These figures-of-merit indicators are most useful to evaluate and compare the optimal trade-off between transparency and sheet resistance for different TCEs^24,28^ given a specific application^23^. By using a manufacturing-grade photonic curing engine (Novacentrix, PulseForge Invent), we now seek to explore the optimal post-processing conditions required to enable the introduction of this silver nanowire-based TCE technologies into state-of-the-art printed electronics manufacturing lines.

### Summary of this work

This report describes how we take advantage of simulation and careful control of the high-speed photonic curing of an interconnected network of silver nanowires, in order to manufacture the best high-performance transparent conductive electrodes. Using standard commercial ITO as our comparison benchmark, we achieve a 2.6–2.7 $\times$ performance gain over ITO using two distinct figure-of-merit indicators. Under optimal conditions, these electrodes can reach transparencies over 90% and sheet resistance values down to 9.8 $\Omega /{\text{sq.}}$, compared to 30 $\Omega /{\text{sq.}}$ for standard ITO. While their performance equals the best results achieved with high-temperature pressure-rolling^9,30,32^, this photonic curing process is also compatible with low-temperature substrates and with the stringent requirements of high-speed roll-to-roll manufacturing lines^45,46^. As such, we firmly believe this low-cost transparent conductive electrode fabrication process using high-speed photonic sintering will greatly facilitate the large-scale manufacturing of multiple new disruptive technologies including low-cost printed solar cells, lighting, haptics, wearable electronics and biomedical sensors.

## Results and discussion

**Figure 1:**
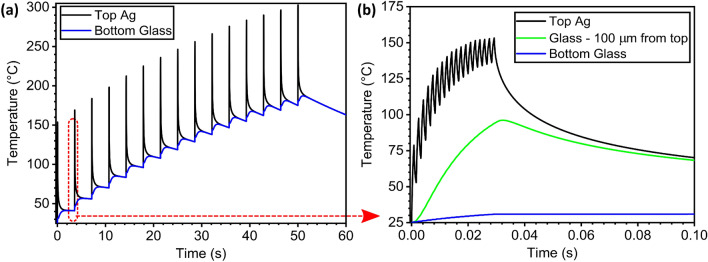
SimPulse simulation of the pulse-train used to process AgNW TCE’s in glass in the Pulseforge (**a**) 15 consecutive pulses. The red oval points to a detailed version of the first pulse in (**b**) Simulation of the effects of a single pulse. For comparison, the green line depicts the simulated temperature profile at 100 $\upmu$m under the top surface of the glass substrate and the blue line shows the simulated temperature at the bottom surface.

In Fig. 1, we show the Pulseforge simulation results of the optimal pulse configuration used to process our TCE samples on glass. Our typical pulse has a fluence of 23.4 J cm$^{-2}$, with an envelope duration of 30,000 $\upmu$s. This irradiation is delivered using a sequence of 15 consecutive pulses to complete the photonic treatment of the film. As shown in Fig. 1a the process brings the film’s temperature to 300 $^\circ$C, a well established temperature value for a proper welding of silver nanowires in thermally-annealed TCE’s^28^. If we look more closely, Fig. 1b reveals how each of these fifteen (15) 30,000 $\upmu$s-long pulses is really made of 18 shorter $\upmu$-pulses with a duty cycle set at 50%.

As a reference, in Fig. 1 we show the temperatures reached in the glass substrate at 100 $\upmu$m under the top surface (green curve) and at the bottom surface (blue curve), in order to properly illustrate the heat dissipation process taking place during this photonic processing. As expected, those temperatures are too low to cause damage in most low-temperature substrates currently used in flexible electronics.

Previous works by Bansal and Malhotra discuss in detail the consequences of intense pulsed-light sintering on nanoparticle necking and the overall temperature profile in metal nanoparticle-based films, both theoretically and experimentally^36^. They describe how the temperature in the films reaches a maximum before cooling down due to increased heat dissipation of the joined nanoparticles as a whole. This phenomenon occurs as a result of the plasmon resonance-induced heating and subsequent welding described by their coupled model^36^. As such, we can confidently conclude, the Novacentrix SimPulse physical modelling tool is closer to their non-coupled model and does not account for this inter-particle necking effect during the film processing. Our basis for reaching this conclusion is fully described in the supplementary material. This first-order approximation can be perfectly acceptable for most industrial processing applications, as long as the users remain aware of this model limitation. Still, the better heat dissipation by the processed film (tailing-off) is already a satisfactory indicator of the correct welding of the nanostructures and the model can serve as a rough indicator of the steady-state temperatures reached in our system. While the PulseForge Invent is capable of delivering higher pulse fluence that could potentially further reduce significantly the processing time, it remains essential to carefully avoid extreme peak temperatures that can damage both the functional material and the substrate. Carefully avoiding these situations is the key to a successful photonic annealing, thus achieving the maximum peak temperature in the thin-film with the lowest substrate temperature possible thanks to the rapid thermal equilibrium reached between the thin-film and the substrate’s surface^33,34^. Indeed, carefully-controlled pulse shaping includes dividing the main pulse into several $\mu$-pulses that help deliver the pulse energy in more specific ways to prevent burning or blowing off the material while reaching the desired temperature goal^33,34^. In the end, the good agreement between the literature results and our SimPulse predictions shown in Supplementary Fig. S-1, are enough to make us highly confident about its capability to analyze the overall behavior of our TCEs under photonic curing conditions.

### Optical and electrical properties of the TCE’s

#### Optical characteristics

**Figure 2:**
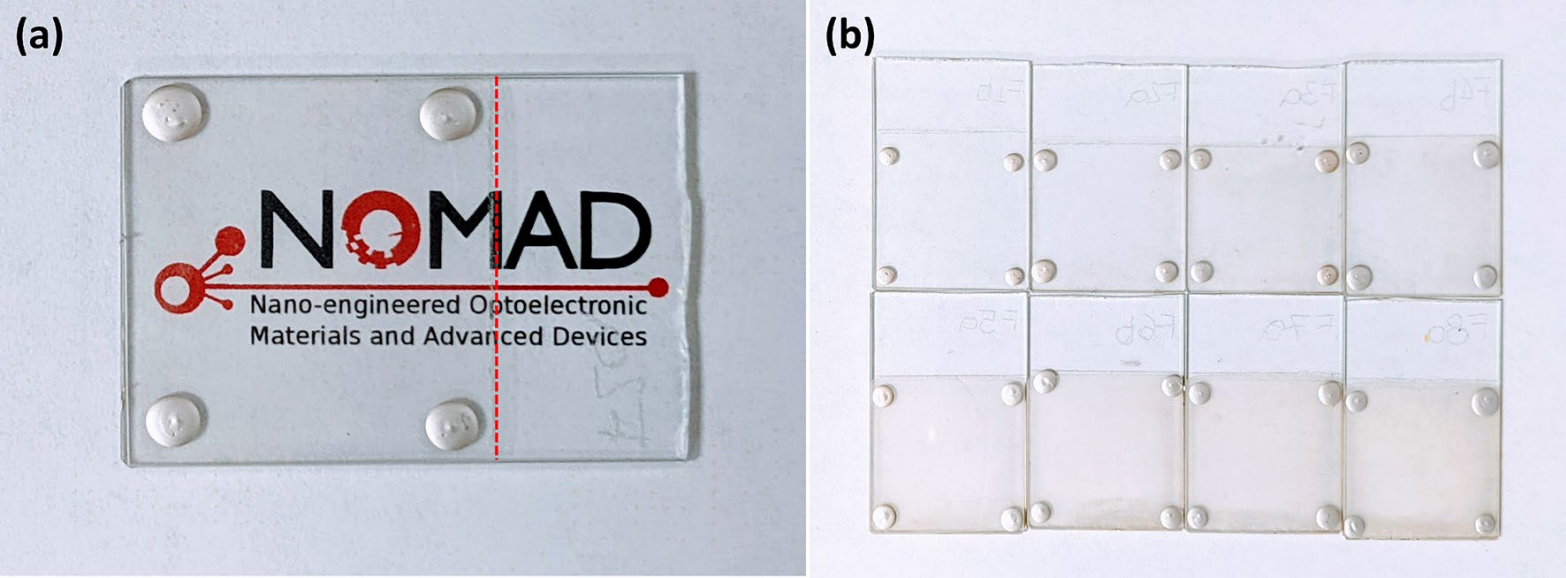
TCE electrodes on glass after photonic annealing. (**a**) Two-layer sample on a piece of paper to highlight its good transparency. The red dashed line shows where the film ends (**b**) Samples showing an increasing number of silver nanowire layers and how their transparency is progressively reduced.

Visual inspection of the samples before and after photonic sintering shows no significant changes in transparency or film quality. Photograph of a typical sample with a double layer of silver nanowires after photonic curing is shown in Fig. 2a, highlighting how the logo can be easily distinguished through the glass and the TCE layer. Photonic treated samples with a range of layers also maintain integrity upon visual inspection as shown in Fig. 2b.

**Figure 3:**
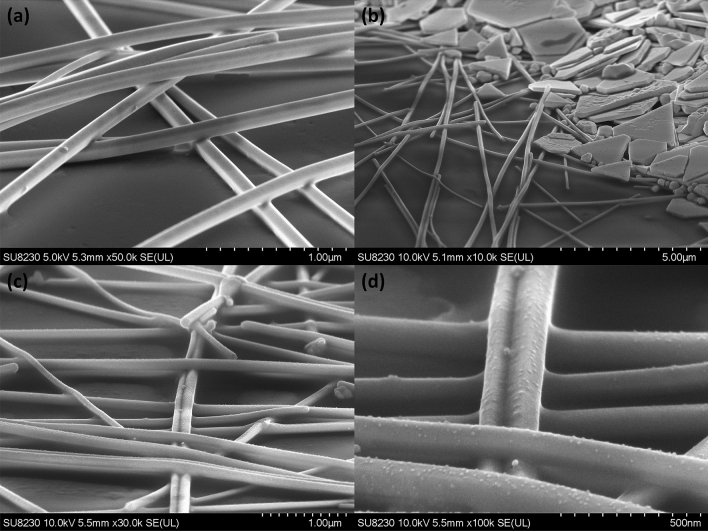
SEM images of the nanowires before and after photonic treatment (**a**) Pristine untreated nanowire films (**b**) Photonic-treated films showing the interface with the silver flakes from the electrical contacts (**c**, **d**) High-resolution micrographs to highlight the successful interconnection of the nanowires yielding better electrical properties after photonic curing.

Scanning electron microscopy (SEM) analysis shown in Fig. 3 allows to compare the morphology of the nanowire-based electrodes before and after photonic curing. Figure 3a exposes how the nanowires maintain their original shape and texture before photonic curing, as well as avoiding potential spherodization of the nanowires^47^. Visual artifacts of the SEM imaging technique gives an appearance of joint nanowires, but in reality this film conducts poorly due to the presence of the PVP coating surrounding the nanowires^22^. Figure 3b suggests that the application and low-temperature treatment of the paste does not affect the integrity of the treated film and there is good physical contact with the nanowire network. After photonic curing treatment, the high-resolution SEM images in Fig. 3c, d reveal morphologies that are expected from a highly-conductive, transparent, thin-film of silver nanowires where deformations resulting from welding between the nanowires can be observed^41^. The nano-textured surfaces for the nanowires as revealed in Fig. 3d appear as a result of the removal of the PVP layer as previously described in the literature^22^.

#### Transmittance and surface coverage fill factor

**Figure 4:**
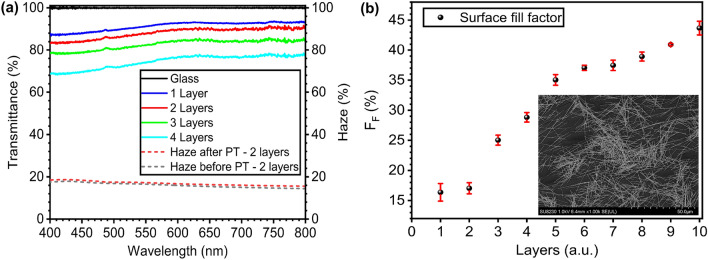
Optical transmittance and haze of the TCEs (**a**) Transmittance spectrum for samples with 1–4 nanowire layers (left axis). The haze characteristics of the sample with 2 layers before and after photonic treatment (dashed lines—right axis) (**b**) Surface coverage fill factor F$_F$ from 1 to 10 layers of deposited nanowires. Inset: SEM image of a 2-layer sample.

These electrodes are all fabricated using a solution of 10 mg/mL of silver nanowires in ethanol, with lengths ranging from 20 to 30 $\upmu$m and an average diameter of 120 nm. The optical transmittance evolution as a function of the number of layers deposited by dip coating is presented in Fig. 4a for samples with 1–4 layers, and using pristine glass as the baseline. Averaging the TCE transmittance values over the visible spectrum (400–800 nm), we obtain 91.5%, 88.3%, 82.8% and 74.7% for samples with 1, 2, 3 and 4 nanowire layers respectively. Haze is another optical parameter that provides information about the light scattering in the film and points to the utility of a TCE in diverse applications. Haze values lower than 1% make TCE’s very useful for display applications whereas higher values highlight their utility in photovoltaic devices^48^. Our champion sample made with 2 layers, presents an average haze over the visible range of 16% before photonic treatment and 16.9% after, shown in Fig. 4a. Even with the change in NW’s texture after treatment shown in Fig. 3d, its haze remains close to its initial value and demonstrates potential use in photovoltaic devices. Finally, the surface coverage fill factor (F$_F$) evolution with an increasing number of layers is shown in Fig. 4b. The values of F$_F$ for samples with number of layers 1–4 are 16.4%, 17.0%, 25.0% and 28.8% respectively.

#### Optoelectronic characteristics

**Figure 5:**
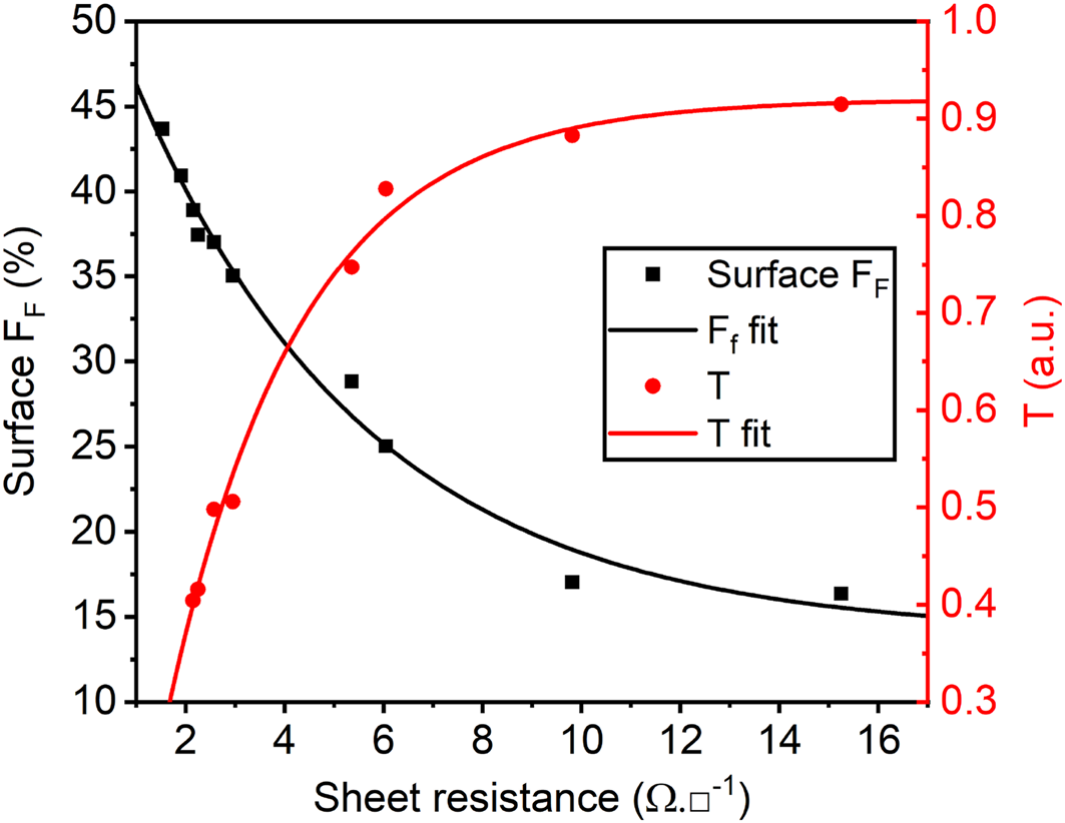
Surface fill factor (F$_F$) and transmittance (T) as a function of the sheet resistance (R$_{sh}$) values for electrodes with varying number of layers. The smooth lines correspond to the nonlinear fit functions.

The values for transmittance (T), surface fill factor (F$_F$), sheet resistance (R$_{sh}$) and Non-Uniformity Factor (NUF)^49^ were obtained following the procedures described in the methods section. In Fig. 5 the evolution of T and F$_F$ are shown as a function of the measured R$_{sh}$. Based on the simulation results (Fig. 1), the values of sheet resistance can be obtained using the established pulse conditions and number of pulses for photonic treatment of the samples. The sheet resistance values thus achieved for the samples with 1–5 layers are 15.3, 9.8, 6.1, 5.4 and 3.0 $\Omega /{\text{sq.}}$ respectively. *NUF* for our champion electrode was calculated from 12 regions in the sample and resulted in NUF = 26%. This value highlights an innate disadvantage of the dip-coating fabrication method, regardless of its low-cost and ease of processing. Other fabrication methods, like Meyer rod method^49,50^ yield better uniformity but were unavailable at the moment of fabrication of our samples.

For TCEs, the transparency of the film usually refers to the transmittance and can be related to R$_{sh}$ using the form $T=e^{-\alpha /\sigma _{DC,B}R_{sh}}$. There, $\upalpha$ represents the absorption coefficient and $\upsigma _{DC,B}$ is the bulk DC conductivity of the film^43^. By fitting the experimental data from Fig. 5, we obtain the empirical relation for T as a function of R$_{sh}$:

1 $$\begin{aligned} T=-1.15\cdot e^{-{0.39}/{1.05}\cdot R_{sh}}+0.92 \end{aligned}$$

where the fitting parameters $\upalpha =0.3906$ cm$^{-1}$, $\upsigma _{DC,B}=1.051$S and the values $-1.155$ and 0.92 represent scaling and translation factors. The $R^2$ value for Eq. (1) is 0.99. As expected, smaller nanowire fill factor result in higher optical transmittance and higher sheet resistance values. In turn, reducing the sheet resistance requires increasing the coverage density, thus reducing the optical transmittance. Expressing Eq. (1) in the form R$_{sh}$ = f(T) yields the following empirical relation:

2 $$\begin{aligned} R_{sh}=-2.69\cdot ln\left( \frac{0.92-T}{1.15}\right) \end{aligned}$$

which can now be used to calculate our electrodes figures of merit.

### Figures-of-merit for the transparent conductive electrodes compared with conventional ITO

When evaluating the performance of transparent conducting electrodes, it is important be able to qualify their characteristics against known variables. Established figures-of-merit (FoM) indicators^24,51^ help establish the optimal trade-off between the transparency T and the sheet resistance R$_{sh}$ and achieve meaningful comparisons.

#### Figure of merit: electrical to optical conductivity ratio $\left( \sigma _{DC}/\sigma _{OP}\right)$

The first figures-of-merit (FoM) indicator looks at the ratio between the electrical and optical conductivity $\left( \sigma _{DC}/\sigma _{OP}\right)$. The value used for the electrical conductivity $\upsigma _{DC}$ represents the bulk DC conductivity of the TCE film. In contrast, the optical conductivity defined as $\upsigma _{OP}$ can be seen as the ratio between the optical absorption coefficient $\upalpha$ and the impedance for free space, $Z_0=120\pi$. As such, one can use the relationship established^24,43^ between the transmittance value T with the sheet resistance values R$_{sh}$:

3 $$\begin{aligned} T=\left( 1+\frac{Z_0}{2\cdot R_{sh}}\frac{\sigma _{OP}}{\sigma _{DC}}\right) ^{-2} \end{aligned}$$

To derive the following expression for the FoM indicator, $\upsigma _{DC}$/$\upsigma _{OP}$:

4 $$\begin{aligned} \frac{\sigma _{DC}}{\sigma _{OP}}=\frac{188.5}{R_{sh}\cdot \left( T^{-{1}/{2}}-1\right) } \end{aligned}$$

As expected, achieving the highest ratio $\upsigma _{DC}$/$\upsigma _{OP}$ requires both high electrical conductivity (meaning low R$_{sh}$ values), coupled with low optical transmittance (meaning high T values). By using the empirical relationship obtained for our TCE electrodes in Eq. (2) and substituting for R$_{sh}$ into Eq. (4), the relationship for this FoM indicator only as a function of T for our TCE electrodes can be re-expressed as:

5 $$\begin{aligned} \frac{\sigma _{DC}}{\sigma _{OP}}=\frac{-70.07}{ln\left( 0.8-\frac{T}{1.155}\right) \cdot \left( T^{-{1}/{2}}-1\right) } \end{aligned}$$

**Figure 6:**
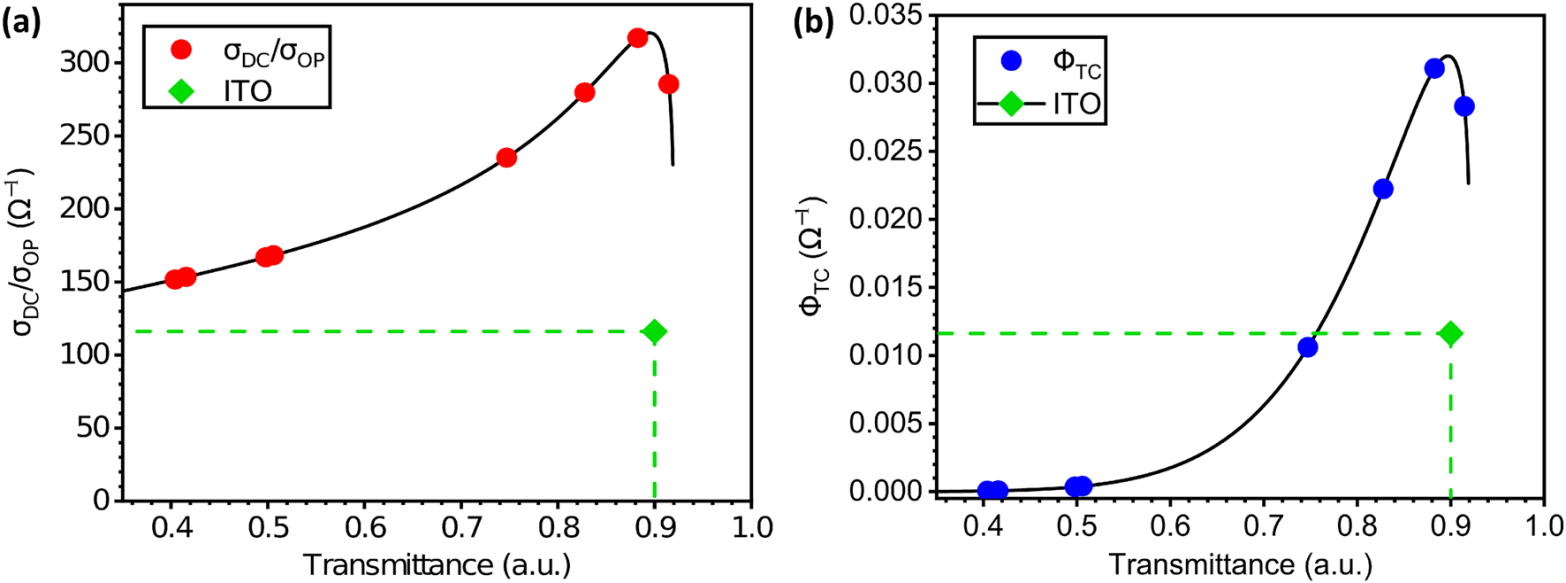
Figures of merit for transparent conductive electrodes (**a**) ${\sigma _{DC}}/{\sigma _{OP}}$ and (**b**) $\Phi _{TC}$. Both figures of merit conclude that the sample with 2 layers offers the best balance between transparency (T = 88.27%) and sheet resistance (R$_{sh}$ = 9.81 $\Omega /{\text{sq.}}$). The FoM values for ITO on glass are represented by the green diamond for reference.

Using this semi-empirical expression, it is now possible to establish the FoM values for our TCE devices, which are shown in the Fig. 6. The maximum TCE performance of $\sigma _{DC}/\sigma _{OP}=317\ \Omega ^{-1}$, is reached with 2 layers of silver nanowires (T = 88.3%, and Rsh = 9.8 $\Omega /{\text{sq.}}$). As seen in Fig. 6a, this FoM value is 2.67$\times$ higher than a conventional glass-covered ITO substrate. This is mostly due to a significantly lower sheet resistance value (T = 90.2%, and Rsh = 30 $\Omega /{\text{sq.}}$), corresponding to a $\sigma _{DC}/\sigma _{OP}=118.7\ \Omega ^{-1}$ obtained using Eq. (5)^14^.

#### Figure of merit: transparent conductive materials $\Phi _{TC}$

A different FoM indicator aims at quantifying and comparing the performance of transparent conductive electrodes with different thickness, sheet resistance and transparency values. To do so, Haacke proposed the following figure of merit indicator^44^:

6 $$\begin{aligned} \Phi _{TC}=\frac{T^{10}}{R_{sh}} \end{aligned}$$

It is important to mention that this $\upphi _{TC}$ is not dimensionless, as one might expect from a conventional figure-of-merit indicator. Here again, we can use the empirical relationship obtained for our TCE electrodes [Eq. (2)], to substitute R$_{sh}$ into Eq. (6). In doing so, this second FoM indicator as a function of T for our TCE electrodes can be described as:

7 $$\begin{aligned} \Phi _{TC}=\frac{T^{10}}{-2.69\cdot ln\left( \frac{0.92-T}{1.155}\right) } \end{aligned}$$

It is obvious from both Eqs. (6) and (7) that this second FoM indicator will greatly favor the TCEs transparency (high T values) over their conductivity (low R$_{sh}$ values). Figure 6b compares this second FoM indicator for our TCE electrodes and for the standard ITO-coated glass.

Once again, the maximum FoM value is found near T=90%, and corresponds to the 2-layer TCE sample (T = 88.27%, and R$_{sh}$ = 9.81 $\Omega /{\text{sq.}}$). The corresponding FoM value is $\Phi _{TC}=0.031\ \Omega ^{-1}$. In contrast, accepted values^14^ for commercial ITO (T = 90.2%, and Rsh = 30 $\Omega /{\text{sq.}}$) correspond to a $\Phi _{TC}=0.012\ \Omega ^{-1}$. As expected, this FoM indicator is 2.58$\times$ higher than ITO for our 2-layer TCE electrode, which is nearly identical to the previous FoM enhancement value (2.67 $\times$).

#### Figure of merit for photonic treatment versus sheet resistance of TCEs, $\Phi _{PT}$

So far, both FoM indicators are based on the relationship between optical transmittance and sheet resistance values. The transmittance is of course defined as the fraction of the incident light intensity that reaches the opposite side (varying between 0 and 1) and it also relates to both the film’s absorption coefficient $\upalpha$ and its thickness *t* using the Beer-Lambert law of absorption to obtain $T=I/I_0=e^{-\alpha \cdot t}$.

**Figure 7:**
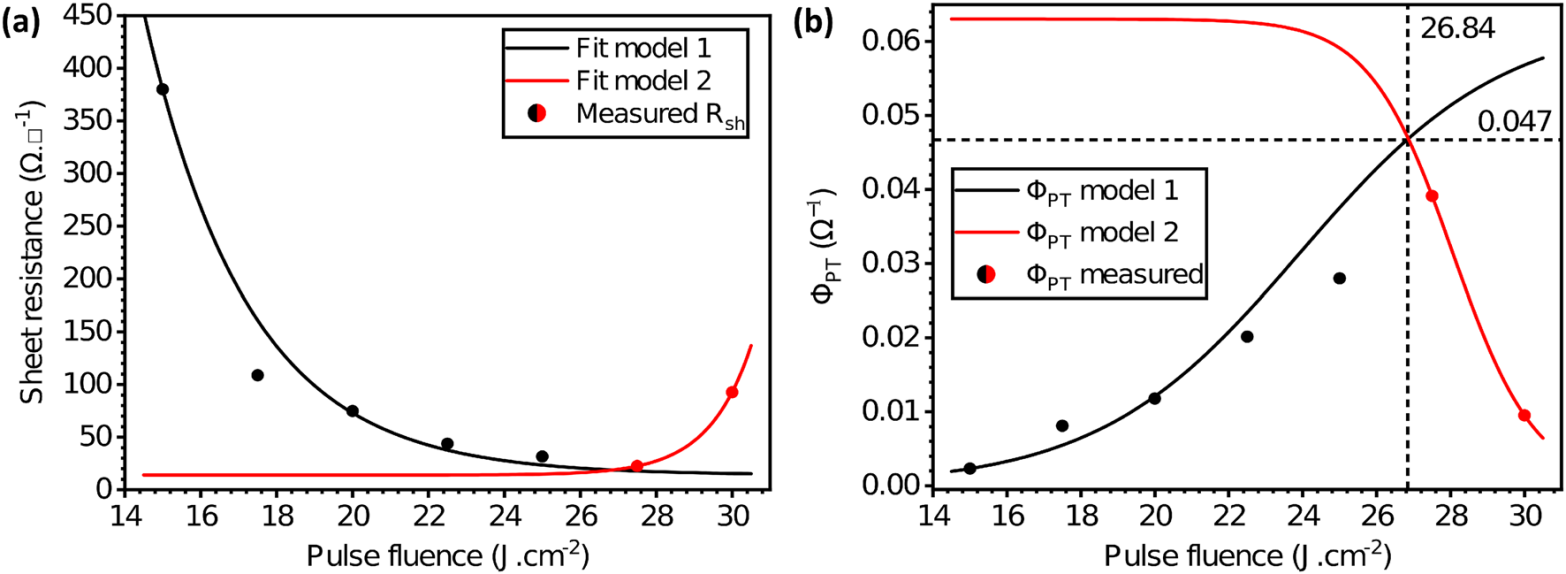
Figure-of-merit redefined for photonic treatment of transparent conductive electrodes $\Phi _{PT}$. Dashed lines mark the highest value of the FoM corresponding to 0.0535 $\Omega ^{-1}$ and a pulse fluence of $F_p=26.4$ J cm$^{-2}$.

Meanwhile, the sheet resistance, $R_{sh}=1/\sigma t$ measures the resistance of a squared area, regardless of the area of the square and it is expressed in terms of the conductivity of the film $\upsigma$ and the film’s thickness *t*. As such, the ratio T/R$_{sh}$ is maximized when the thickness $t = 1/\alpha$ and T = 0.37. This value of transparency does not sit well with TCE’s and it is why Haacke redefined the FoM giving more weight to the transmittance T using the general form^44^ $\Phi _{TC}=T^x/R_{sh}$.

Similarly to the first two FoM indicators, we can now propose a new figure-of-merit indicator for photonic curing that relates the sheet resistance values of the TCE film with the pulse fluence $F_p$ (instead of T). First an empirical relation between $F_p$ and R$_{sh}$ can be derived from the plot in Fig. 7a. Given the evolution of R$_{sh}$ at higher values of $F_p$ (introducing light-induced damage), it becomes necessary to split the fitting function into two models that will represent a specific set of values (black and red circles in Fig. 7). The obtained models are represented in Eqs. (8) (below the damage threshold) and (9) (above the damage threshold).

8 $$\begin{aligned} R_{sh}^{(1)}&= 15+360\cdot e^{\left( -0.4\cdot \left( F_P-15\right) \right) } \end{aligned}$$

9 $$\begin{aligned} R_{sh}^{(2)}&= 15+1\cdot e^{\left( 0.88\cdot \left( F_P-25\right) \right) } \end{aligned}$$

Based on our preliminary results, we define this figure-of-merit for photonic treatment of transparent conductive electrodes as $\Phi _{PT}=1/R_{sh}$. Incorporating the empirical Eqs. (8) and (9) into this new expression for $\Phi _{PT}$ yields the FoM Eqs. (10) and (11):

10 $$\begin{aligned} \Phi _{PT}^{\left( 1\right) }&= \frac{1}{R_{sh}^{\left( 1\right) }}\ =\ \frac{1}{15+360\cdot e^{\left( -0.4\cdot \left( F_P-15\right) \right) }} \end{aligned}$$

11 $$\begin{aligned} \Phi _{PT}^{\left( 2\right) }&= \frac{1}{R_{sh}^{\left( 2\right) }}\ =\ \frac{1}{15+1\cdot e^{\left( 0.88\cdot \left( F_P-25\right) \right) }} \end{aligned}$$

This non-dimensionless semi-empirical FoM indicator’s evolution as a function of the pulse fluence is shown in Fig. 7a for samples with 2 layers of silver nanowires. The maximum value for this FoM is $\sigma _{PT}=0.0535\ \Omega ^{-1}$ with a $F_p=26.4$ J cm$^{-2}$. As previously explained, we chose a pulse fluence value of 24.5 J cm$^{-2}$, which gives a $\sigma _{PT}=0.0364\ \Omega ^{-1}$ for the sake of speed and ease of processing. Just like for the thermal annealing of silver nanowire-based electrodes, a maximum performance is reached before the electrode starts to degrade due to light-induced damage. Certainly, different factors can affect this FoM indicator, which is also based on empirical results as the other two established indicators. However, we believe it is consistent and further supports the previous conclusions drawn from the SimPulse simulations. In industrial production lines, we believe great benefits can arise from quickly characterizing and predicting the optimal fluence conditions for a given process using this indicator in conjunction with the SimPulse industrial simulation toolboxes.

## Conclusions

In this work, we successfully produce high-performance transparent conductive electrodes (TCEs) made of a silver nanowire meshes on glass substrates. The photonic sintering post-processing speeds are compatible with industrial manufacturing equipment used for printed electronics. The silver nanowire films are deposited using carefully-controlled dip-coating to precisely control the nanowire densities and processed using light-pulses from a state-of-the-art manufacturing-grade photonic curing machine (Novacentrix, PulseForge Invent). These electrodes display performances akin to thermally-annealed silver nanowire-based electrodes.

We first use SimPulse simulations and compare with known results available on the literature (for validation). While the SimPulse simulation results are consistent with the literature, we observe it does not account for the interaction between nanoparticles (inter-particle necking). As long as this model limitation is well understood, the simulation tool is accurate, and we conclude that it can be used for rapid optimization of the photonic treatment parameters for industrial-grade manufacturing purposes. We then use this simulation tool to estimate the optimal curing parameters for our nanowire electrodes.

Observing the nanowire morphology before and after the photonic curing process, we confirm that the photonic sintering significantly benefits from the metallic nanowires’ morphology. The radiation energy delivered by the lamp is efficiently absorbed through surface plasmon resonance, rapidly increasing their temperature sintering the wires into a highly-conductive interconnected nanowire network.

Looking at our TCEs properties, we find that two (2) deposited layers of nanowires yield the best balance between transmittance (88.3%) and sheet resistance (9.8 $\Omega /{\text{sq.}}$) values. As expected, the same sample exhibited the best performance when analyzed using the two most common figures-of-merit indicators for TCEs, namely $\sigma _{DC}/\sigma _{OP}=317.1\ \Omega ^{-1}$ and $\Phi _{TC}=0.031\ \Omega ^{-1}$. These performance values are 2.6–2.7 $\times$ higher than commercial-grade ITO glass, the reference for commercial transparent conductive electrodes.

Finally, we propose a new figure-of-merit indicator for photonic curing that describes the balance between pulse fluence and sheet resistance for a constant transparency value. We observe that the photonic treatment of the silver nanowire films exhibits a bi-exponential behavior originating from the light-induced damage from exposure to excessive fluence. These results can be replicated at lower pulse fluence by incrementing the number of pulse repetitions and the emission frequency. This however, may not be convenient in fast-paced industrial manufacturing environments but it leaves room for further investigation. We show that the optimal fluence value of 24.5 J cm$^{-2}$ (experimental) is consistent with the theoretical value of 23.9 J cm$^{-2}$ predicted by this FoM ($\Phi _{PT}$) model. We believe using this figures of merit presented here, will help the community to better understand and predict the metallic nanowire photonic treatment phenomenon.

We believe that the results presented in this paper may help to accelerate the development of printed electronics technology. Particularly, in the rapid fabrication and integration of high-performance silver nanowire-based transparent conductive electrodes in multiple opto-electronic, energy-harvesting and sensing applications.

## Methods

### TCE deposition

A dispersion of silver nanowires in ethanol is purchased from ACS material chemical supplier. The average nanowire diameter is 120 nm and their lengths range from 20 to 30 $\upmu$m. The nanowire dilution is adjusted to a concentration of 10 mg/ml and then used as is. Commercial glass substrates with dimensions 1 $\times$ 1.5 inches are obtained from Micro-mechanics. The glass slides are cleaned according to the following protocol: First, thorough washing with alconox detergent and rinsing with deionized water. Second, 15 min sonication in isopropanol. Finally, 15 min sonication in acetone. The silver nanowire mesh electrodes are deposited by dip-coating. The nanowire solution is placed in a beaker of appropriate size and the glass slides are coated using an immersion still time of 5 s and the withdrawal velocity was set to 200 mm/min. The holder height is adjusted so that the nanowire-covered surface area is 1 sq. inch. Kapton tape is used to cover the back of the glass substrates to prevent covering of both sides of the glass.

### Photonic annealing of the samples

Each sample is processed using the Novacentrix PulseForge Invent system. The samples are taped to the system tray and only the area covered by the silver nanowires is exposed to the light pulses. To do so, the uncovered top section of the glass is covered by a metal plate (mask) held by magnets embedded in the tray. The pulse conditions, number of pulses and exposure method were kept the same for all samples. Only the number of layers in the sample fabrication is changed to vary the nanowire density (and the TCE transparency). Using a single flash, we optimized the pulse fluence in order to achieve the lowest sheet resistance values for TCE samples with 2 layers of silver nanowires. The lowest sheet resistance was obtained with a pulse fluence of 27.5 J cm$^{-2}$ with the single pulse characteristics presented in Fig. 1b. At this fluence value, the emission frequency of the machine is limited and the processing time increases. For this reason we chose a slightly lower fluence at 24.5 J cm$^{-2}$ to enable faster processing, achieving low sheet resistance while reaching the target temperature of 300 $^\circ$C as predicted in our simulations from Fig. 1a. From the simulation, the number of pulses to reach 300 $^\circ$C is set to 15 and is the value used for processing all the samples. After photonic processing, commercial silver-flake paste (Novacentrix, Metalon HPS-021LV) is used to apply the electrical contacts on the four corners of the silver nanowire films by drop casting a small amount and curing at 80 $^\circ$C for 60 min. This paste facilitated measuring the electrical conductivity of the films without damaging or affecting the film’s acquired electrical properties in any way.

### Sample characterization

After photonic curing and drying of the the silver paste pads, the sheet resistance is measured with a Keithley 2400 source unit to measure the resistivity values across the contacts. The non-uniformity factor (NUF) was done dividing the total area of the sample in 12 regions and measuring the sheet resistance on each region using a Four-point Probe system from Ossila. The calculation of the NUF value was done following the procedure described elsewhere^49^ Nanowire surface fill factor F$_F$ was measured using a 3D laser confocal microscope LEXT OLS4100 from Olympus. Ten 120 by 120 $\upmu$m images are taken at random places in the films, using the 100X objective. For each image, the auto-enhance setting from the microscope’s own software is applied to improve clarity and contrast. Afterwards all images were binarized using Matlab, where white pixels corresponded to nanowires while black pixels to areas devoid of them. The surface fill factor F$_F$ is then computed by dividing the number of white pixels over the total pixel count of the image. Finally, the value for F$_F$ of each of the ten images taken per sample are averaged to obtain the overall F$_F$ value for a given sample. Scanning electron microscopy (SEM) characterization is performed using a SU8230 microscope from Hitachi. Transmittance T was measured using a OceanOptics Flame spectrometer and a LS-1 tungsten halogen lamp that covers the full visible spectrum from 400 to 800 nm. The 100% transmittance baseline is set using a clean glass slide to obtain only the transmittance value for the TCE. The film’s transmittance value is obtained by dividing the measured transmittance over the glass transmittance at that respective wavelength (baseline correction), then averaged over the whole spectrum (400–800 nm). Finally, these ten transmittance values are again averaged to provide the final transmittance value for any given sample. Haze was measured using the UV–VIS–NIR spectrophotometer Perkin Elmer, Lambda 750 with an integrating sphere. This measure required four scans, two of them with the sample located at the entrance of the integrating sphere providing the total transmittance (T2) and the forward scattered light of the sample (T4). The other two scans were done without the sample to remove the scattering of light originated in the equipment itself, with one scan using the white standard (T1) and one open-ended (T3). Haze is then calculated with the following relation: $Haze=(T4/T2-T3/T1)\cdot 100\%$.

## Supplementary Information

Supplementary Information 1.
