# Supplementary material for: High-performance silver nanowires transparent conductive electrodes fabricated using manufacturing-ready high-speed photonic sinterization solutions
Source: Sci Rep. 2021 Dec 17;11:24156. doi: 10.1038/s41598-021-03528-w (PMC8683411; doi:10.1038/s41598-021-03528-w)
Supplement: Supplementary file 1 — Supplementary Information 1. [file 41598_2021_3528_MOESM1_ESM.pdf]

High-performance silver nanowires transparent conductive electrodes fabricated using manufacturing-ready high-speed photonic sinterization solutions.

Luis Felipe Gerlein   Jaime Alberto Benavides-Guerrero   Sylvain G. Cloutier*

SUPPLEMENTARY INFORMATION

Validation of the SimPulse simulations

This detailed comparison intends to validate the SimPulse simulation tool and its application for the post-processing treatment of our silver nanowires thin-film electrodes. This commercial integrated photonic curing simulation tool is intended to reduce processing times in industrial environments. The sample's material stack can be created with their corresponding thicknesses to match real conditions as closely as possible. It yields the appropriate processing parameters for photonic curing and sintering a variety of commercial metallic nanoparticles inks and slurries, widely employed in the printed electronics world. Here, we compared the SimPulse model's prediction with reports from the literature.

Indeed, Bansal et *al.* and Dexter et *al.* already provided a comprehensive framework to elucidate the behavior of nanowires under intense pulse light sintering[1,2]. Based on their experimental parameters and results, we can replicate the same thermal behavior using the SimPulse tool. To do so, one must set the simulator parameters in order to match the pulse fluence conditions reported and adjust the machine parameters within the inherent speed limitations of the machine. The results shown in Figure S-1(a-c) depict the behavior predicted by SimPulse tool using the same pulse fluence conditions as previously reported[1,2]. The material stack is configured to be the same, from top to bottom: 20 nm layer of silver, 250 μm of polyimide substrate, 1500 μm of borosilicate glass buffer. The pulse settings were set as follows: single pulse duration 5000 μs, duty cycle of 50%, pulse frequency of 3 Hz, no micro-pulses.

We then extended the pulse number from 150 (shown in Figure S-1(a-c) dashed lines) used in the literature[1,2], to 1000 pulses to achieve a steady state temperature matching the top surface's temperature predicted by the non-coupled model already analyzed. Also, our pulse emission frequency is different at these fluence conditions, a factor that affects the heating rate of the metallic nanowires. Aiming to match the thermal behavior of our simulations to those previously reported[1,2], E3 in this case, we changed the voltage applied to the lamp diode and adjusting for pulse frequency, the fluence required to obtain a peak temperature around 300 °C after 150 pulses is 3.49 J.cm^{-2}, as shown in Figure S-1(d). Interestingly, the delta of temperature within the Kapton layer from top to bottom oscillates around 100 °C, according to the simulations. Because of the rapid cooling between layers, this does not affect the substrate.

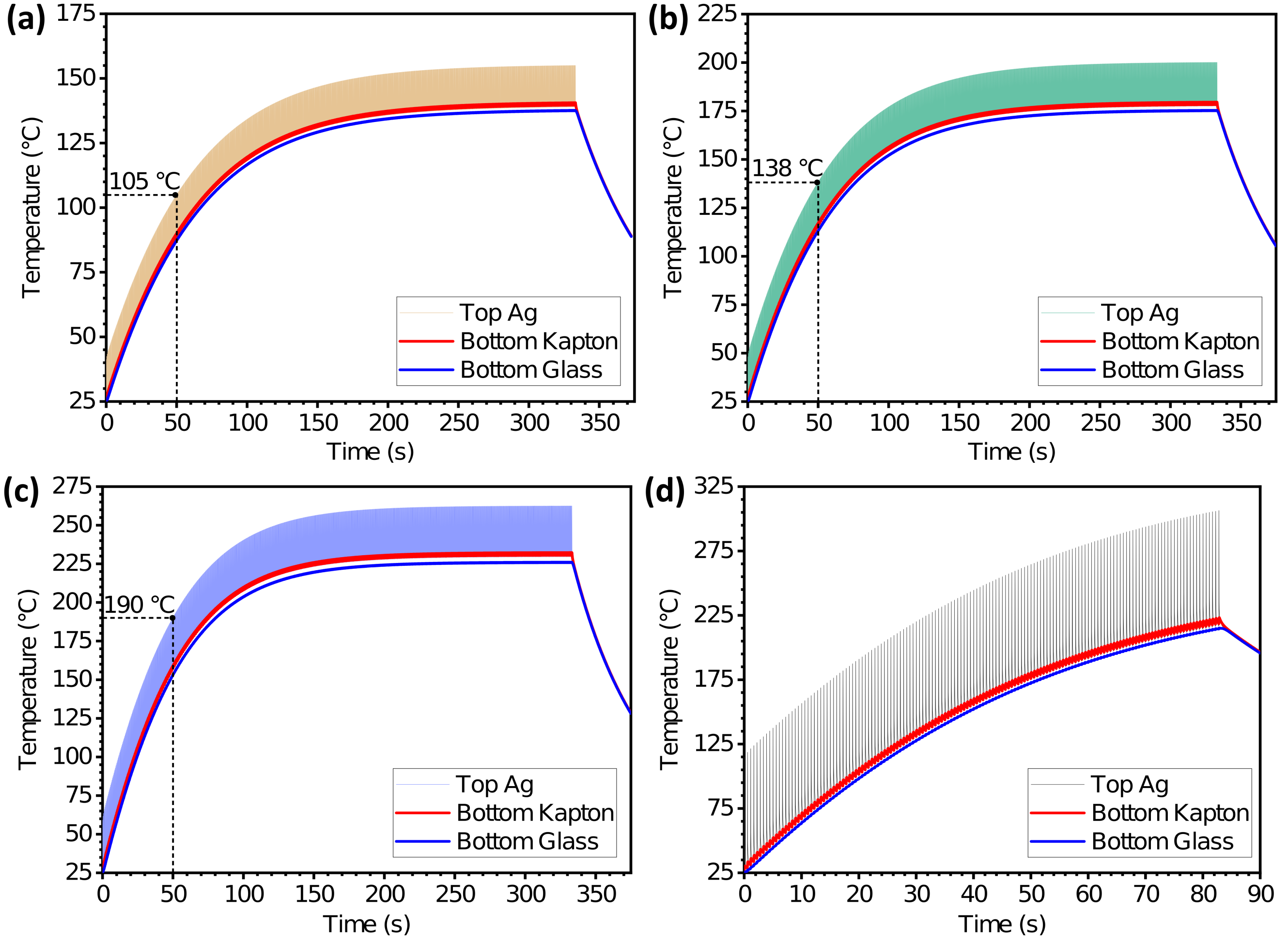

Figure S-1. Validation of the Simpulse simulation for nanoparticles. SimPulse temperature predictions using 1000 pulses to match the results reported by Bansal et al. at different energy densities[1]: (a) E1=0.85 J.cm^{-2}, (b) E2=1.2 J.cm^{-2} and (c) E3=1.8 J.cm^{-2}. The dashed lines represent the 150th pulse count and the respective temperature reached up to that point (d) Simulated pulse conditions matching the thermal behavior of E3 with 150 pulses

References

1. Bansal, S. & Malhotra, R. Nanoscale-shape-mediated coupling between temperature and densification in intense pulsed light sintering. *Nanotechnology* **27**, 495602, DOI: https://doi.org/10.1088/0957-4484/27/49/495602 (2016).
2. Dexter, M., Bhandari, R., Chang, C.-H. & Malhotra, R. Controlling processing temperatures and self-limiting behaviour in intense pulsed sintering by tailoring nanomaterial shape distribution. *RSC Adv.* **7**, 56395–56405, DOI: https://doi.org/10.1039/C7RA11013H (2017).
